# Making a decision about surgery for female urinary incontinence: a qualitative study of women’s views

**DOI:** 10.1007/s00192-020-04383-5

**Published:** 2020-06-29

**Authors:** Rebecca Lynch, Philip Toozs-Hobson, Jonathan Duckett, Douglas Tincello, Simon Cohn

**Affiliations:** 1 Department of Health Services Research and Policy, Faculty of Public Health and Policy, London School of Hygiene & Tropical Medicine, London, UK; 2 School of Population Health and Environmental Sciences, Faculty of Life Sciences and Medicine, King’s College London, Addison House, Guy’s Campus, Great Maze Pond, London, WC1H 9SH UK; 3 Birmingham Women’s and Children’s NHS Foundation Trust, Birmingham, UK; 4 Medway NHS Foundation Trust, Gillingham, Kent UK; 5 Department of Health Sciences, College of Life Sciences, University of Leicester, Leicester, UK

**Keywords:** Decision-making, Surgery, Urinary incontinence, women’s views, Surgical mesh

## Abstract

**Introduction and hypothesis:**

This qualitative interview study explores aspects women with urinary incontinence(UI) reflect upon when considering whether or not to have surgery. Conducted prior to the recent mesh pause in the UK, the article provides insights for current and future approaches to shared decision-making.

**Methods:**

Qualitative in-depth interviews of 28 patients referred to secondary care for stress and mixed UI who were considering UI surgery. Participants were recruited from four urogynaecology clinics in the Midlands and South England, UK. Interviews were conducted in clinics, in patient homes, and by telephone. Data analysis was based on the constant comparative method.

**Results:**

Participants’ accounts comprised three key concerns: their experience of symptoms, the extent to which these impacted a variety of social roles and demands, and overcoming embarrassment. Accounts drew on individual circumstances, values, and concerns rather than objective or measurable criteria. In combination, these dimensions constituted a personal assessment of the severity of their UI and hence framed the extent to which women prioritized addressing their condition.

**Conclusions:**

Acknowledging women’s personal accounts of UI shifts the concept of ‘severity’ beyond a medical definition to include what is important to patients themselves. Decision-making around elective surgery must endeavour to link medical information with women’s own experiences and personal criteria, which often change in priority over time. We propose that this research provides insight into how the controversy around the use of mesh in the UK emerged. This study also suggests ways in which facilitating shared decision-making should be conducted in future.

## Introduction

The recent controversy in the UK over the use of surgical mesh to treat female urinary incontinence (UI) has led the National Institute for Healthcare Excellence (NICE)—the body dedicated to inform the National Health Service over appropriate, and affordable, treatment options—to produce a decision tool for women considering the procedure. This aid comprises plain English summaries of the latest clinical evidence concerning possible complications to help women reflect and order their priorities around symptom control and possible risks [1].

This patient decision aid, and others like it, can clearly play a vital role [2]. However, women’s decisions are not necessarily based solely on their appreciation of clinical evidence or assessing the physical risks an intervention might have for them individually. Rather than conceiving patient decisions as being based solely on medical understandings, and a quest for certainty, this article draws on qualitative data to examine what other factors women draw on when faced with decisions around UI surgery.

The promotion of shared decision-making has long been established as good practice within the NHS and beyond [3]. While urgent or emergency treatments rarely constitute occasions for patients to be actively involved in decisions, long-term treatments and elective surgery both present opportunities for them to deliberate their options [4, 5]. Much has been written about how and when patients might be able to truly participate [6–9]. However, there has also been critique [10], especially concerning the undeniable differences in expertise and authority between clinicians and patients [11]. It is therefore pertinent to question whether providing just clinical evidence is ever an adequate strategy to ensure patients genuinely engage in a decision-making process. This is a particularly important consideration in relation to a stigmatized condition such as UI, which has multiple cultural values, meanings, and associations.

To address these, we present a qualitative study that was conducted *prior* to the national pause on the use of mesh for pelvic floor reconstruction and the publication of the NICE decision aid. The timing of data collection means that we are able to highlight other factors which are just as pertinent to women when considering whether to have surgery or not, but which tend to be omitted in the traditional decision-making paradigm. Specifically, medical consultations base decision-making discussions on clinical measures of UI severity, namely the frequency and quantity of urine leakage [12]. However, this may not be the most meaningful or relevant criteria for women themselves. This article presents the ways women themselves judge the severity of their condition, its priority in their lives, and the criteria they draw on to make decisions about surgery.

## Materials and methods

Semi-structured interviews were undertaken with women considering surgery for their UI. Participants were recruited from four urogynaecology outpatient clinics in different parts of England: Birmingham, Gillingham (Kent), Leicester, and Southampton, between May–December 2017. Female patients referred to these clinics with stress or mixed incontinence who met the study criteria (considering UI surgery, > 18 years old, no history of urology surgery) were approached by members of the clinic teams (including JD, DT, PTH and physiotherapists, nurses, surgical consultants, and specialist registrars) as part of routine appointments. Those potentially interested in participating were given an information sheet and form to complete and return directly to the social science research team at LSHTM (RL and SC). Members of clinical teams were not informed which patients had returned forms. Interviewees were then purposively sampled by region and age to ensure a diverse, rather than statistically relevant, sample.

Interviews covered women’s experiences of UI, actions they took to accommodate the condition, and their expectations of future surgical/non-surgical interventions. Interviews were conducted face to face or by phone, according to patient preference. Written consent was always obtained after participants were given the opportunity to ask clarifying questions. Interviews were based on a general topic guide, lasted between 40 and 130 min, and were audio-recorded. Transcription was undertaken verbatim by an external company accustomed to dealing with confidential data. Publicity relating to the mesh controversy grew in England and Wales during the study, but interviews were completed several months before this specific intervention was put on ‘pause’ while further evidence was called for [13].

Transcripts were anonymized and coded using the qualitative data management system NVivo 10. Following a constant comparative method [14, 15], emerging themes were followed up in greater depth during subsequent interviews, such that the topic guide became focused on subjects that were most important to participants themselves. Initial coding was undertaken by RL while recruitment and interviews were underway. The codes, drawn both deductively from the interview topic guide and inductively through early analysis, were augmented and refined during analysis. SC oversaw the process and blind-coded a sample to ensure reliability. Codes were arranged into hierarchies, with those deemed to be too similar collapsed together; the resulting structure was discussed with JD, DT, and PTH who helped further refine the analysis.

The study was funded by the UK National Institute for Health Research (NIHR) Health Services and Delivery Research (HS&DR) Programme (14/70/162). Patient representatives from the UK’s Royal College of Obstetricians and Gynaecologists (RCOG) Women’s Voices network (an online panel of members of the public who have experiences of using women’s health services) were consulted when designing the study and applying for funding and remained involved as members of the project’s advisory board. They informed the design of the interview topic guide and data analysis through discussion and feedback of early findings and conclusions drawn.

## Results

Twenty-eight female patients were interviewed from across the four sites. The overall age range was 26–74 years (see Table 1). Fourteen requested to be interviewed in their homes, 11 in clinics, and 3 by telephone. There were no significant differences across the four sites, despite predictions that they were drawn from distinct populations. Most participants had had UI for an extended period of time; in some cases, for > 20 years. For all the women, surgery was clinically indicated and an immanent possibility.

**Table 1:** Participant characteristics

Participant characteristics	*n*
Age (years)	
20–39	6
40–49	4
50–59	7
60–69	6
70 and over	5
Total women interviewed	28
Clinic attended	
Birmingham	14
Gillingham (Kent)	7
Leicester	7
Southampton	1

Speaking to patients who were actively thinking about surgical options allowed exploration of those factors women themselves considered and raised as important, which were often related to their personal experiences of UI and how they had lived with it up until this point. Rather than being led by a decision tool that forefronted clinical evidence, this approach alerted us to broader values and understandings women had and how these connected to their everyday lives. Women’s considerations centered on three key concerns: perceptions of how serious the physical symptoms of the condition was; how to prioritize their condition and its treatment over a wide range of responsibilities that they had in their daily life; and the need to overcome embarrassment and shame as their condition became more visible through seeking further treatment.

### ‘How bad is my condition?’

Rather than adopting an abstract clinical measure of severity, women often tried to assess their condition by comparing themselves to other women with similar symptoms or of similar age. In this way they attempted to establish what a ‘normal’ amount and frequency of urine loss might be;

‘I’ve had the same peer group since I was a young girl, and my friends will go out and they don’t have to take spare knickers in their bag in case they laugh on the dancefloor or they trip over, or a sudden movement happens that they have an issue with incontinence. (Interviewee 11, 38 years old)

Such comparisons were then linked to the possible treatment routes they were considering;

‘…my problem doesn’t feel bad enough to go for surgery…I really wanted to keep going with the physio and try and sort it out that way … (Interviewee 7, 51 years old)

Trying to establish if their UI was particularly bad sometimes also included reflections on the likely burden of certain interventions on NHS resources. This led women to question whether their current symptoms were sufficiently severe to warrant financial justification, especially when they might just be part of ageing;

‘… do I take any action around this, is this a normal part of ageing, do I accept it, do other people accept it, is the problem I’ve got worse, or not as bad as other people? You don’t know how to compare yourself with other people because it’s not really talked about… I mentioned it to two people and they’ve both said, well they implied, that it’s something you just accept with age.’ (Interviewee 6, 66 years old)

However, a common catalyst to consider surgery for the women in this study was not how ‘bad’ their symptoms were in some objective way, but, instead, how much ‘worse’ they had become over time. As their personal circumstances changed, many reported how their symptoms became more obvious and unmanageable;

‘I feel like it’s got worse, I feel like my bladder has got weaker, that I haven’t been able to retain it as much…’ (Interviewee 26, 32 years old)

The changing nature of the symptoms, and the feeling that they could no longer be successfully managed, shaped the way in which the option to have surgery gained likelihood. But while some described having surgery in terms of an investment in their future, this was often hard for many interviewees to establish definitively;

‘I don’t really want it [the operation]. But if I delay it, I’m getting older, whereas obviously, 40, almost 41, my youngest is 13, my oldest is 17, so at some point they’re going to leave. I might as well enjoy the time now, and be able to do more.’ (Interviewee 23, 40 years old)

Overall, judging how bad their condition was as a basis of whether to have surgery was always a complex and personal process. Rather than striving to establish a consistent independent measure, women tended to adopt a comparative approach to their evaluations—whether in relation to other women that they spoke to or read about, their assumptions about what an ageing woman’s body might be like, or by comparing their current symptoms with personal recollections concerning how their UI symptoms had been in the past. This more subjective, relational strategy reflects the fact that the women invariably talked about their condition using specific real-life experiences and detailed first-hand accounts rather than a detached and depersonalized medical assessment of their UI.

### Competing demands

In addition to trying to describe the seriousness of their condition in terms of other women or the progression of symptoms, participants invariably described management of UI and the possibility of surgery in relation to competing demands with their role as mothers, daughters, partners, siblings, friends, and employees;

‘It doesn’t just affect me because it affects my partner as well because when we go out…if we're out for an hour then we have to come back. So, it affects him, you know…or if I go out with friends, or whatever, I end up having to come home.’ (Interviewee 28, 47 years old)

These social roles were clearly central not only to how the women thought about themselves, but crucially also judged the relative significance of their UI and importance of their treatment. One participant, for example, talked about having to cancel one of her diagnostic tests as she had to attend to her daughter that day, while another prioritized caring for her 8 year old leading her to opt for injections rather than the more invasive and time-demanding surgery.

All the women described strategies they had adopted not to let their UI disrupt these roles as far as possible; for example, by always carrying (and hiding) incontinence pads with them; by having changes of clothes at hand, or wearing dark trousers to hide any accidents; or avoiding particular activities, such as going running or playing with their children or grandchildren;

‘The most difficult and tough thing about it, was that when you want to go and do something, it’s like, come on the bouncy castle, or come and do this or do that, you sort of think, no I can’t do it… ‘cause I was leaking’ (Interviewee 20, 51 years old).

Core to all these everyday tactics was the attempt to accommodate and hide their UI, frequently conveying a tension between their commitment to different forms of work and care they did for others and the personal impact of their UI symptoms. Women whose symptoms were very pronounced and impacted their lives in a dramatic way found it easier to consider a surgical option. For others, it was the fact that these kinds of demands had reduced over the years which meant that they were now considering surgery; for example, one patient in her 60s said that now that her husband’s ill health had got better, she could finally attend to her own condition. It is also worth noting that for some women, UI was not their only health condition and dealing with other health issues sometimes competed with it or were more urgent to resolve through treatment.

Although a minority of interviewees talked about feeling too embarrassed to share their situation with anyone they knew, others discussed their situation with partners, friends, colleagues, and sometimes parents or daughters, in addition to with clinical staff. These informal conversations helped them reflect on the impact of their UI on their everyday lives, the extent to which it should potentially take precedence, and hence whether to decide to have surgery. Key to these more casual social interactions was that, rather than trying to gain greater medical understanding, they tended to include the emotional and personal dimensions of having UI and what they hoped surgery might change.

### Impact of embarrassment and shame

Embarrassment and shame were key elements of most women’s accounts of living with UI, but crucially these feelings had wider reverberations in relation to the decision about treatment options. Many participants said that although they had tried to find out about other women’s personal experiences, the stigmatized nature of the condition meant that intimate details were not often discussed or shared openly. As a result, the women described how they had to glean information indirectly; they described how they would ‘listen out’ for relevant comments from the casual conversations of different people they encountered as well as learning things in an ad hoc fashion from newspapers, magazine articles, and websites that they stumbled across.

While women described how, in the past, they had put much time and effort into hiding their condition effectively, seeking further treatment required making their UI more visible. Participants explained the difficulty they had in overcoming their feelings and finding the right words and appropriate way to talk about their experiences to clinical staff. Although the gender of the staff member was significant to some, who invariably preferred speaking to women, more generally it was the manner of the clinician and the fact that the problem was taken seriously that emerged as more significant. Follow-up appointments were often easier to have than the initial time these concerns had to be dealt with;

‘I think I was probably more comfortable going back again, because I’d been there before, I remembered him, he was a lovely doctor, and I guess the intrusion had already been done, I’d already opened up, you know what it’s like, the first times you speak about some things are harder’ (Interviewee 21, 46 years old)

However, not only were women having to disclose the state of their symptoms to health professionals, but they often also had to describe their UI, at a new level of detail, to significant others. Having to have tests, go to appointments, and perhaps ultimately have surgery inevitably meant they had to talk more openly to spouses, family, friends, or employers to work out arrangements for their current roles, responsibilities, and work commitments. But this meant that their UI inescapably would become more public.

As for clinical staff, female friends, family members, and work associates were viewed as easier to speak to than male acquaintances;

‘My husband, I did tell him a bit, but you don’t need to tell him too much, he’s a man. Yes, I won’t go into depth with him… Obviously, my friends don’t need to know, and the children, I will [tell] them at some point. The boys, I won’t tell as much as my daughter, she would have a bit more information, because the boys won’t be bothered’ (Interviewee 23, 40 years old)

Finally, the possibility of a surgical intervention further exacerbated these concerns over shame and embarrassment for many, with many expressing a fear that the procedures inevitably would mean that an intimate part of their anatomy would have to be exposed;

‘I didn’t think they would actually be doing it through the vagina. So, obviously anything to do with your private area you’re going to be a bit nervous about. It’s something completely different that, the only person that’s ever been there is when I’ve had children.’ (Interviewee 10, 26 years old)

Overall, these personal feelings of shame and embarrassment presented an initial obstacle that participants had to overcome to seek help once they felt their UI symptoms had become intolerable: What had often been very private and secret for many years had to be made more public and visible. However, these feelings also remained an ongoing aspect that shaped how women engaged with any future treatment choices and the kinds of interactions that would inevitably be part of the process. In this way, the very emotive dimensions of UI were not simply a secondary response to the condition, but had a direct effect on the options considered and the consequences future interventions would have on a wide variety of their social relationships.

## Discussion

Although it may be assumed that the greater the clinical evaluation of UI severity the more essential women would regard having surgery, this study suggests this is often not a straightforward relationship; many more values, understandings, and experiences are brought into consideration. While medical decisions about the suitability of patients for UI surgery frequently assess severity through the quantity and frequency of leakage, women judge the severity of their UI using a much broader set of criteria, ranging from personal bodily experiences to how extensively the condition disrupts their social roles and everyday routines. As part of this process, women routinely draw on the ideas, opinions, and experiences of others. As such, if the concept of ‘severity’ is to be drawn on as part of a shared decision-making process, it must move beyond a medical assessment of UI to include what is important to women themselves. Based on our findings, two different women with the same level of frequency and amount of unintentional urine loss can experience their UI very differently. Severity is consequently always contingent, and identical interventions are not the same or desired solution for different patients, even if clinically indicated as being so.

There is no doubt that decision aids can be very useful and help patients understand and appreciate complex and often competing clinical factors [2]. However, our study makes it clear how women do not evaluate surgical options ‘in the abstract’, according to epidemiological ideas of risk or so-called objective measures. Instead, they draw on particular, everyday experiences to weigh up factors and guide them. The result is not only that thinking about options routinely draws on a diverse set of other people and elements, but also that it can respond to changing values and priorities, as different experiences, hopes, and expectations become foregrounded. Decisions are therefore rarely made conclusively; some degree of hesitancy, uncertainty, and indeterminacy is always present. By broadening our understanding of the range of relevant values that women draw on, we argue that decision-making around surgery is not only a distributed process based on multiple criteria, but that factors often take on greater importance than others at different times.

Additionally, while medical accounts often depict decisions as singular events based on rational, knowledge-driven processes, our findings demonstrate how, in practice, many decisions are actually established over multiple meetings with different people and encounters [16, 17]. Many of these encounters are shaped by emotional aspects of the condition, as much as simply discussing options and exchanging knowledge. In particular, the need to shift from strategies of secrecy to having to be more open and public about their UI not only meant women frequently hesitated, but also re-evaluated whether their symptoms really were so intolerable after all.

In combination, we suggest that these findings potentially offer insight into how the controversy over the use of mesh took off in the UK. Rather than simply arising from competing claims over medical evidence, doubts about the procedure gained veracity through media representations of women’s first-hand accounts [18]. These had a level of influence precisely because they served as a vehicle to amplify many of existing emotional and experiential dimensions that we describe in this article.

A limitation of the study is that in only recruiting patients who had been referred to secondary care and were considering surgery, we were unable to capture those women whose experiences of UI and thoughts around surgery meant that they remained within primary care or were not using NHS services at all. Furthermore, women who did not wish, or felt unable, to be interviewed may share characteristics that were not captured, especially as the study was unable to sample according to ethnicity. Investigating the experiences of women who had had successive urogynaecology surgeries was beyond the realm of this study but it is likely that previous surgeries for similar symptoms may produce a different range of responses than those considering this kind of intervention for the first time. While age differences are evident in the responses given by the women we interviewed, a greater number of interviews may well have revealed further segmentation of the participant cohort, potentially highlighting differences both due to biological age or because they were brought up and socialized during very different eras.

Despite such limitations, our study suggests that in considering patient concerns around the use of mesh in surgery, clinical staff should not overly focus on factual inaccuracies that patients may have, but look to understand the wider context of patients’ lives to recognize that this is often just as active a domain of considerations in their decision-making process. Indeed, promoting clinical evidence as a response to the controversy without doing so has the potential to further disenfranchise many patients who seek a wider sense of meaningful dialogue and support. We therefore suggest that any future iteration of a patient decision-aid, such as that produced by NICE, should integrate the non-clinical aspects we describe in addition to current clinical evidence, since these social and emotional factors will always remain central to how medical information is interpreted and contextualized by women.

These considerations also have important consequences for thinking about personalizing clinical approaches to decision-making more generally and potentially provide insight into mechanisms by which some medical controversies and conspiracies take hold. Acknowledging the fact that patients rarely, if ever, base their assessments solely on clinical symptoms, their understanding of medical evidence, and the likelihood of a successful intervention according to current scientific evidence demands approaches that genuinely take into account their own points of view and the relationships between a condition and their wider social networks, circumstances, roles, and situations.
